# Personalized ctDNA micro-panels can monitor and predict clinical outcomes for patients with triple-negative breast cancer

**DOI:** 10.1038/s41598-022-20928-8

**Published:** 2022-10-22

**Authors:** Erica K. Barnell, Bryan Fisk, Zachary L. Skidmore, Kelsy C. Cotto, Anamika Basu, Aparna Anand, Megan M. Richters, Jingqin Luo, Catrina Fronick, Meenakshi Anurag, Robert Fulton, Matthew J. Ellis, Obi L. Griffith, Malachi Griffith, Foluso O. Ademuyiwa

**Affiliations:** 1 Department of Medicine, Division of Oncology, Washington University School of Medicine, 660 S. Euclid Ave., St. Louis, MO 63110 USA; 2 McDonnell Genome Institute, Washington University School of Medicine, St. Louis, MO USA; 3 Siteman Cancer Center, Washington University School of Medicine, St. Louis, MO USA; 4 Division of Public Health Sciences, Department of Surgery, Washington University School of Medicine, St. Louis, MO USA; 5 Lester and Sue Smith Breast Center, Dan L. Duncan Comprehensive Cancer Center, Baylor College of Medicine, Houston, TX 77030 USA; 6 Department of Genetics, Washington University School of Medicine, St. Louis, MO USA

**Keywords:** Breast cancer, Tumour biomarkers

## Abstract

Circulating tumor DNA (ctDNA) in peripheral blood has been used to predict prognosis and therapeutic response for triple-negative breast cancer (TNBC) patients. However, previous approaches typically use large comprehensive panels of genes commonly mutated across all breast cancers. Given the reduction in sequencing costs and decreased turnaround times associated with panel generation, the objective of this study was to assess the use of custom micro-panels for tracking disease and predicting clinical outcomes for patients with TNBC. Paired tumor-normal samples from patients with TNBC were obtained at diagnosis (T0) and whole exome sequencing (WES) was performed to identify somatic variants associated with individual tumors. Custom micro-panels of 4–6 variants were created for each individual enrolled in the study. Peripheral blood was obtained at baseline, during Cycle 1 Day 3, at time of surgery, and in 3–6 month intervals after surgery to assess variant allele fraction (VAF) at different timepoints during disease course. The VAF was compared to clinical outcomes to evaluate the ability of custom micro-panels to predict pathological response, disease-free intervals, and patient relapse. A cohort of 50 individuals were evaluated for up to 48 months post-diagnosis of TNBC. In total, there were 33 patients who did not achieve pathological complete response (pCR) and seven patients developed clinical relapse. For all patients who developed clinical relapse and had peripheral blood obtained ≤ 6 months prior to relapse (*n* = 4), the custom ctDNA micro-panels identified molecular relapse at an average of 4.3 months prior to clinical relapse. The custom ctDNA panel results were moderately associated with pCR such that during disease monitoring, only 11% of patients with pCR had a molecular relapse, whereas 47% of patients without pCR had a molecular relapse (Chi-Square; *p*-value = 0.10). In this study, we show that a custom micro-panel of 4–6 markers can be effectively used to predict outcomes and monitor remission for patients with TNBC. These custom micro-panels show high sensitivity for detecting molecular relapse in advance of clinical relapse. The use of these panels could improve patient outcomes through early detection of relapse with preemptive intervention prior to symptom onset.

## Introduction

Neoadjuvant systemic therapy is widely used in patients with triple-negative breast cancer (TNBC). This therapeutic approach permits assessment of clinically meaningful responses in vivo, which enables rapid identification of effective drugs for tailoring adjuvant systemic therapy^1^. Approximately 30–65% of patients with TNBC who receive neoadjuvant systemic therapy achieve a pathological complete response (pCR)^2–7^. Patients who do not achieve a pCR tend to have a high rate of recurrence and poor overall survival^8–10^. The 3 year risk of distant recurrence for non-pCR patients is 27% versus 9% for those achieving pCR^11^. Three year event-free survival after neoadjuvant chemotherapy is approximately 57%-68% in patients without pCR vs. 92%-94% in patients with pCR^6,8^. The median survival, once TNBC has recurred, is only 18–28 months^12,13^. The inability of chemotherapy to eradicate minimal residual disease is believed to be due to the escape of cells intrinsically resistant to chemotherapy^14^, which are not detectable with the current tools in routine clinical practice.

In this context, a liquid biopsy to measure circulating tumor DNA (ctDNA) offers a promising tool for evaluating real-time response to chemotherapy. In advanced TNBC, next-generation sequencing of ctDNA shows high concordance with mutations seen in tissue biopsies^15^. ctDNA has also been evaluated as a promising tool to predict outcomes in patients with breast cancer^16–22^.

Most traditional sequencing approaches for ctDNA in patients with early-stage TNBC utilize universal panels targeting recurrently mutated genes that are commonly observed in TNBC^21,23^. In these studies, panels do not include somatic variants that are unique to each individual’s tumor. Since TNBC has high patient-to-patient heterogeneity, such universal panels may miss patient-specific molecular changes. As such, we hypothesize that custom micro-panels might improve sensitivity related to measuring clinical outcomes for patients in clinical remission for breast cancer.

There have been a small cohort of studies that have evaluated custom micro-panels for this clinical utility. The c-TRAK TN trial utilized custom ctDNA assays to track 1–2 patient-specific mutations to predict outcomes and intervene with systemic therapy in a moderate-high risk cohort of patients with early-stage TNBC^24^. At a median follow-up of 20.4 months, only 27.3% of patients had detectable ctDNA at 12 months, despite 72% having overt metastatic disease on staging at time of ctDNA detection. These results suggest that custom assays composed of multiple variants may improve the sensitivity of detecting minimal residual disease via ctDNA. Several additional studies have correlated custom ctDNA expression with pathological complete response (pCR)^22,25^, and one study showed correlation between detection of minimal residual disease (MRD) after curative surgery and disease free survival^26^. Most commonly, these studies obtained ctDNA measurements at diagnosis and/or at 1-year post surgery to compare presence of ctDNA biomarkers to ultimate disease status after a disease-free interval. However, tracking custom ctDNA measurements during neoadjuvant therapy, at surgical resection, and at 3–6-month intervals during disease-free intervals might provide additional insight into the prognosis for patients with TNBC.

**Figure 1:**
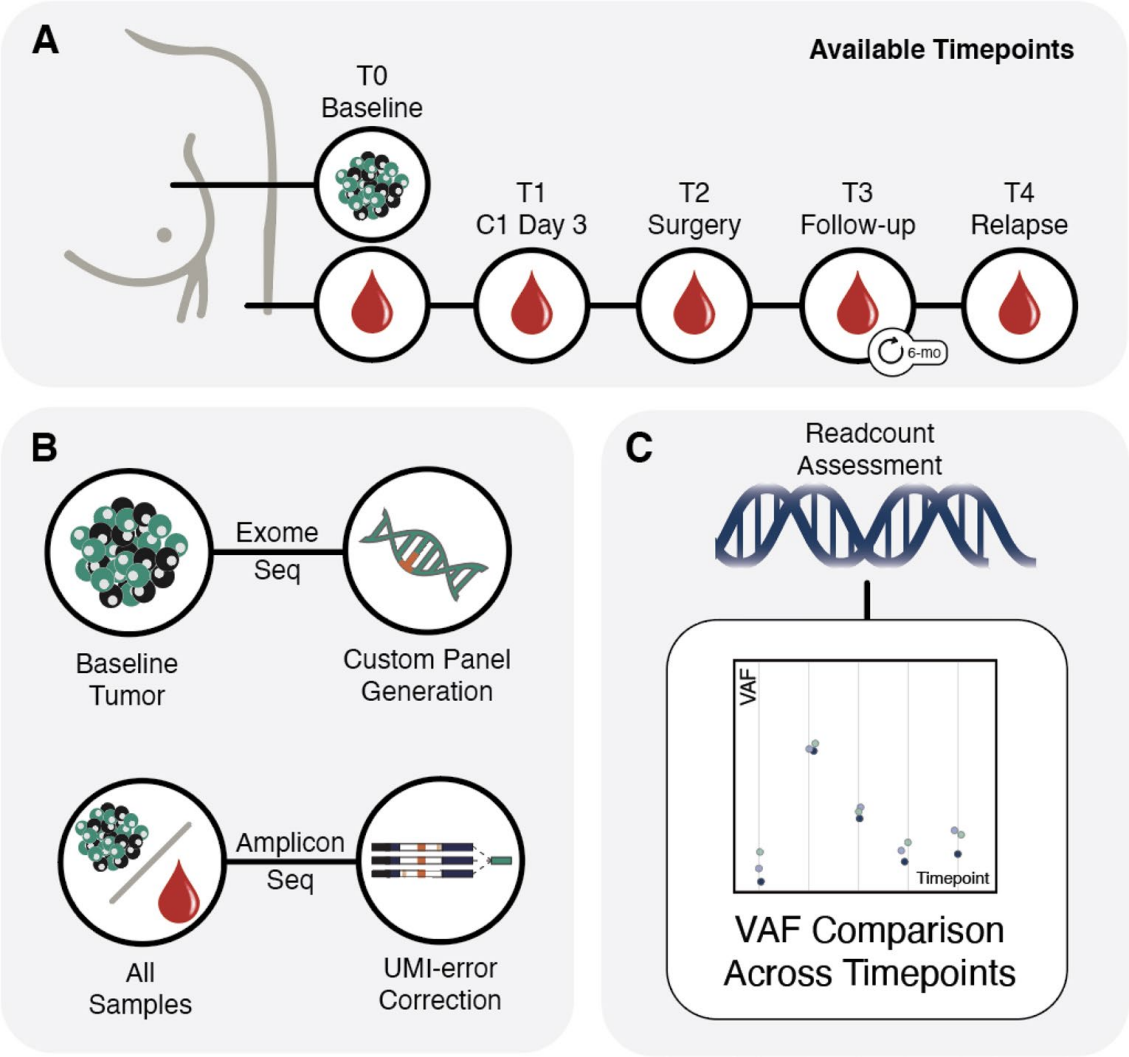
Overview of the methods for sample collection and sequencing. (**A**) Tumor samples from biopsy were obtained at baseline (T0). Blood samples were obtained at baseline (T0), Cycle 1 Day 3 (T1), at time of surgery (T2), during follow-up (T3- every 3–6 month intervals for 5 years), and during relapse (T4). (**B**) Baseline tumor samples were subjected to whole exome sequencing (WES) and variants were identified to build a custom micro-panel. All blood samples (T0–T4) were assessed using the amplicon based custom micro-panel. **C.** Sequencing was subjected to unique molecular identifier (UMI) based error correction. Variant allele fractions (VAFs) were ascertained for baseline biopsies and ctDNA blood samples at all timepoints.

Herein, we report the results of a study of 50 patients in which we sought to determine if longitudinally tracked ctDNA levels before, during, and after chemotherapy could predict clinical outcomes in TNBC patients treated on an ongoing prospective trial (Fig. 1).

## Methods

### Patient clinical trial overview

This study enrolled patients into a phase II de-escalation clinical trial designed to determine if a non-anthracycline regimen will achieve similar rates of pathological complete responses to a standard anthracycline-containing regimen. Eligible patients treated on the parent clinical trial included women at least 18 years old, with estrogen receptor (ER) negative (Allred score < 3 or less than 1% positive staining cells in the invasive component of the tumor) and HER2 negative (0 or 1 + by IHC or FISH negative) invasive breast cancer^27^. Additional eligibility criteria included: Eastern Cooperative Oncology Group Performance Status of 0 to 2, adequate organ and marrow function, and tumor size ≥ 2 cm in one dimension by clinical or radiographic exam (WHO criteria)^28^. Patients with palpable lymph nodes were eligible regardless of tumor size. Exclusion criteria included prior treatment of the current cancer, uncontrolled intercurrent illness, bilateral or inflammatory cancer, pregnant/nursing, or prior sentinel lymph node biopsy. The study was approved by the Institutional Review Board (IRB) at Washington University School of Medicine and followed the Declaration of Helsinki and Good Clinical Practice guidelines. Written informed consent was obtained from each participant in the clinical trial. ClinicalTrials.gov identifier is NCT02124902.

All patients were treated with intravenous docetaxel 75 mg/m2 and carboplatin AUC 6 cycled every 21 days for a total of six cycles. Definitive surgery was performed 3–5 weeks after completion of neoadjuvant chemotherapy. Patients received adjuvant radiation when indicated, and adjuvant chemotherapy for patients without pCR was left to the treating physician’s discretion.

### Sample collection

When possible, tumor biopsies were obtained at baseline prior to chemotherapy (T0), on Cycle 1 Day 3 of chemotherapy (T1- optional), and at the time of definitive surgery following neoadjuvant chemotherapy in patients with residual disease (T2). Matched normal samples (T0-only) were obtained from matched leukocyte germline DNA.

In addition, up to 50 mLs of peripheral blood were collected prospectively from patients at baseline (T0), Cycle 1 Day 3 (T1), at time of definitive surgery (T2), and approximately every 6 months after surgical resection of the breast cancer for a total of 5 years. If applicable, peripheral blood draws were also obtained at the time of relapse (T4). ctDNA was extracted from plasma collected in Streck tubes.

### Sample acquisition

**Figure 2:**
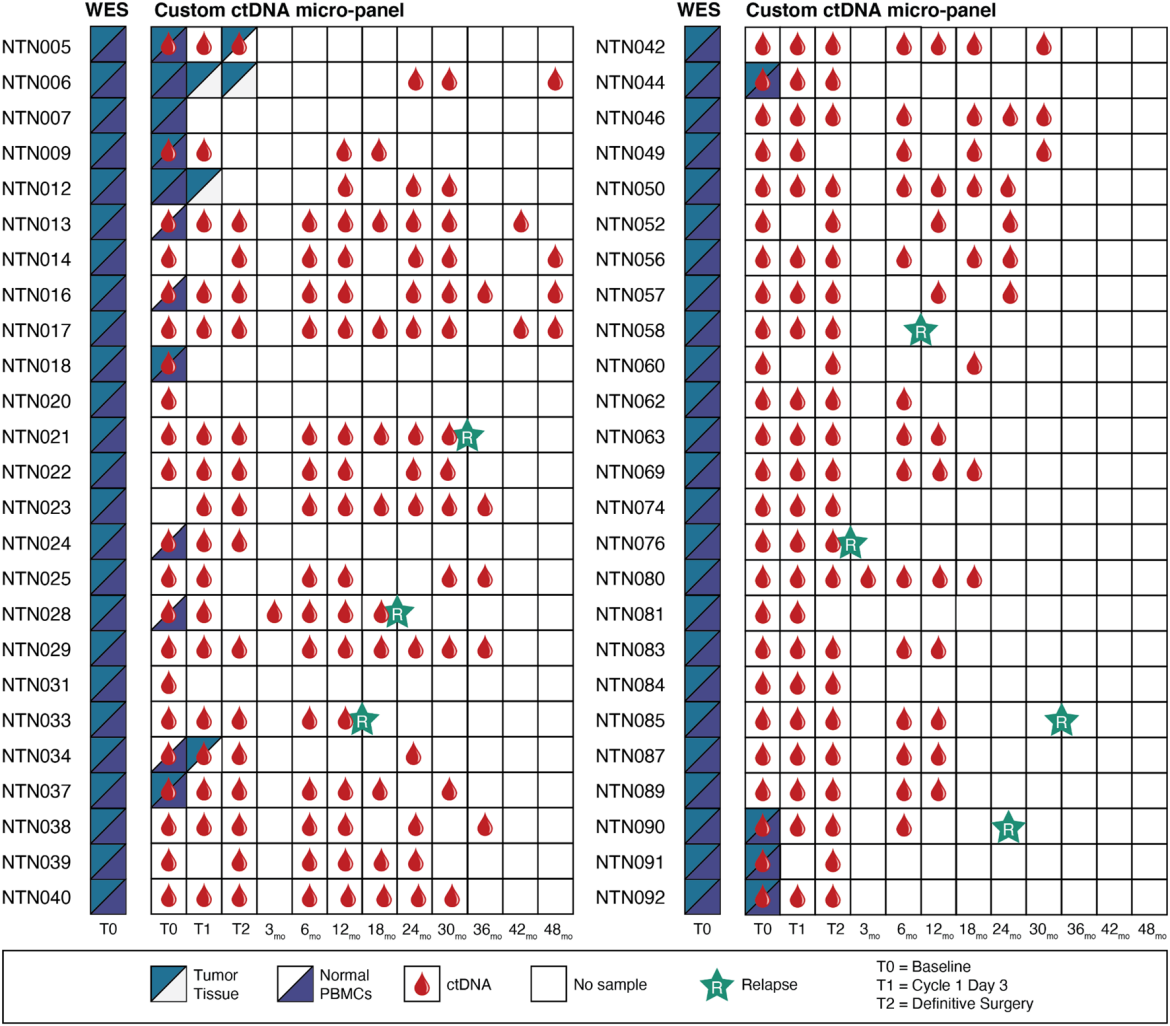
For each of the 50 samples employed in the analysis, two sequencing platforms were employed: whole exome sequencing (WES) and custom ctDNA micro-panel amplicon sequencing. All matched tumor/normal genomics DNA samples underwent WES at baseline (T0). Based on sample availability, micro-panel sequencing was employed on matched tumor/normal genomic DNA samples, tumor-only genomic DNA sample, normal-only genomic DNA sample, or peripheral blood ctDNA samples obtained at various timepoints.

Samples from 50 patients were used for this correlative study. A total of 378 blood samples were obtained and sequenced (Fig. 2). All 50 patients had genomic WES completed for matched tumor/normal pairs at Baseline (T0). Additionally, all 50 patients had ctDNA custom micro-panel sequencing performed on peripheral blood for at least one timepoint during the disease course. ctDNA custom micro-panel sequencing was performed for 46 patients at Baseline (T0), 39 patients at Cycle 1 Day 3, and 40 patients at Surgery. After surgery, follow-up timepoints ranged from 3 to 48 months. More than half of all patients (*n* = 29 patients) had ctDNA custom micro-panel sequencing performed at 6 months and the average patient had 5 timepoints with ctDNA custom micro-panel sequencing (range = 1–10 timepoints). Additional genomic sequencing of tumor tissue and normal peripheral blood mononuclear cells (PBMCs) was performed at Baseline (T0), at Cycle 1 Day 3 (T1), and at Surgery (T2) for 16 patients based on sample availability. In total, 7 patients had clinical relapse observed during the study, 4 of which had blood collected within 6 months of relapse.

### Whole exome sequencing

Tumor DNA was extracted from fresh-frozen biopsies and matched germline DNA from peripheral blood samples. WES data was generated for 50 unique baseline tumors and matched blood DNA samples using the Illumina platform. Paired-end sequence libraries were constructed as described previously^29^ with the following modifications. Samples were barcoded at the ligation step using Illumina unique dual barcodes adapters (Cat# 20,022,370) and were amplified for 6–8 cycles using the Library Amplification Ready-mix containing KAPA HiFi DNA Polymerase (Kapa Biosystems, Inc). For capture enrichment, libraries were pooled in equimolar ratios in groups of 10 and were hybridized in solution to the HGSC VCRome 2.1 design^30^ Enriched libraries were sequenced on the NovaSeq 6000 instrument using the S4 reagent kit (300 cycles) to generate 2 × 150 bp paired-end reads.

### Somatic variant calling from exome data

Raw sequencing reads from tumor and normal tissue were aligned to the human reference genome GRCh38 using Burrows-Wheeler Aligner^31^. The aligner output was merged (if needed) and de-duplicated using Picard MarkDuplicates. A combination of four variant callers (Mutect^32^, Varscan^33^, Strelka^34^, and Pindel^35^) was used to identify somatic variants by comparing normal and tumor variant calls as previously described^36^. Variants were then annotated with the Variant Effect Predictor (VEP) using Ensembl v95 annotations and filtered based on several criteria: previously known variants, variants with high population-specific allele frequency, variants that are based on a high percentage of reads with 0 mapping quality, variants with low depth, and low confidence variants^37^.

### SWIFT custom amplicon assay development

After somatic variant calling, filtering, and manual review of variants^38^ that were detected by exome sequencing (as described above) 4–6 variants were selected for each patient to build a custom ctDNA micro-panel. Variants selected included any non-silent *TP53* variant observed supplemented by up to three additional variants with the highest variant allele fraction (VAF) (see Supplementary Table 1).

ctDNA was isolated from 2 to 10 mL of plasma utilizing two different protocols: (a) the Maxwell RCS instrument and Maxwell RSC ctDNA Plasma Kit (Promega) or (b) QIAamp MinElute ctDNA Mini kit (Qiagen). The isolated ctDNA was dried down to 14 uL prior to a PCR-based enrichment strategy utilizing a custom designed Amplicon HS Panel from Swift Biosciences. Custom micro-panels designed at Swift Biosciences included UMIs and were limited to ~ 100 amplicon primer pairs per pool and were separated into two batches.

Batch 1 was composed of 26 cases which included 154 ctDNA samples (multiple timepoints per case) and the baseline tumor and normal genomic DNA, when available. Batch 2 was composed of 24 cases which included 109 ctDNA samples (multiple timepoints per case) and the baseline tumor and normal genomic DNA, when available. The ctDNA input per enrichment ranged between 1 and 44 ng. Tumor and normal genomic DNA input was consistently 25 ng. The amplicon libraries were constructed as outlined by the Custom Amplicon HS library prep protocol and Target Amplification insert provided by Swift Biosciences, with the following exceptions: automated libraries were generated on the Ep5075 instrument (Eppendorf); bead purification steps were completed with AMPure XP beads at the same ratios outlined in the Swift HS Panel protocol; custom dual unique sample indexes were added per library (TruSeq like primers with 10 bp unique dual indexes, diluted to 2 uM per Swift recommendation); and the final index PCR step was increased to 9 cycles. Different sample types (ctDNA, gDNA from Tumors, and gDNA from Normals) were processed on separate days to prevent any cross-contamination.

The concentration of the final libraries was determined by qPCR (KAPA Biosystems/Roche). 2 × 150 bp paired-end sequence data was generated on the NovaSeq 6000 S4 flow cell targeting ~ 6 million read pairs per library. A unique UMI was assigned to each original single-strand template, corresponding to the first 10 bp of read 2.

### SWIFT sequence data alignment, UMI consensus determination, and VAF estimation

Processing of ultra high depth amplicon sequence data from the SWIFT targeted assay was performed using a computational pipeline that employs custom-built tools created at Washington University. Sequencer generated FASTQ files were first demultiplexed and aligned to the reference genome (GRCh38). During this process, UMI sequences were added to the BAM file using fgbio ExtractUmisFromBam^39^. Once aligned, reads were grouped using fgbio GroupReadsByUmi and collapsed into read families using fgbio CallMolecularConsensusReads. Consensus reads were filtered using fgbio FilterConsensusReads if the read error rate exceeded 5% and individual bases in the consensus were filtered if the error rate exceeded 10%. If the read comprised greater than 50% no calls from this base filtering, then the read was removed. Quality control (QC) measures included requiring a minimum mean unique coverage of 500 reads. Variant allele frequencies (VAFs) were obtained for each somatic variant position (previously detected by exome sequencing as described above) using Bam-readcount^40^.

### Germline variant calling for BRCA Variants

Exome sequencing data from baseline normal samples was used to assess samples for pathogenic or likely pathogenic germline gene mutations for breast cancer. Samples were analyzed with germline CWL pipelines developed at Washington University and variants were called across all genes. *BRCA1* and *BRCA2* variants were identified and annotated using the Variant Effect Predictor tool (VEP)^37^. Variants were manually reviewed using a previously defined standard operating procedure^38^. Variant pathogenicity was evaluated using the ClinVar Allele Registry^41^ and the BRCA Exchange^42^. BRCA status observed on (WES) was compared with previously performed molecular tests to ensure the accuracy of the pipeline.

### Statistics

The overall sample size for the study (*n* = 50) was driven by the need to obtain enough relapse cases so that the lower 95% exact confidence limit for the sensitivity was at least 50%. Assuming a relapse rate of 15–20%, we anticipated 10 patients eligible for the primary analysis. At an observed sensitivity of 90% (9 of 10 samples detected using molecular analysis), the lower bound of the 95% confidence interval was 55%. Reduced relapse rate in our population (*n* = 7), and incomplete compliance with blood collection process (*n* = 25) prevented statistical endpoints from being significant. All data reported in this study should be considered observational. To assess differences between ctDNA positivity and pathological complete response (pCR), a Chi-Square test was used.

## Results

### Clinical Data

Primary clinical outcome results from the phase II parent clinical trial have been published^27^. For the translational cohort in which ctDNA was surveyed longitudinally, the average age was 52 years (range 25–74), 32% were African-American, and 84% had clinical stage II disease. Table 1 details the patient and tumor characteristics for this translational cohort. Seven patients (14%) developed recurrent disease, and 5 patients (10%) had died at the time of report generation.

**Table 1:** Overview of patients and demographic information.

ID	Age	Race	*BRCA* PCR Analysis	*BRCA* WES Analysis*	Stage at Dx	Nodes at Dx	Grade at Dx	pCR	Recurrence	Duration of Follow up (months)
NTN005	50	Caucasian	Negative	Negative	2	Yes	2	No	No	60
NTN006	44	African American	Negative	Negative	3	Yes	3	No	No	67
NTN007	54	African American	Negative	Negative	2	Yes	3	Yes	No	64
NTN009	57	African American	Negative	Negative	2	No	3	No	No	39
NTN012	49	Caucasian	BRCA1	BRCA1	2	No	3	No	No	43
NTN013	64	African American	Negative	Negative	2	No	3	No	No	64
NTN014	50	Caucasian	Unknown	Negative	2	Yes	3	Yes	No	64
NTN016	57	Caucasian	Unknown	Negative	2	Yes	3	Yes	No	64
NTN017	54	Caucasian	Negative	Negative	2	No	3	No	No	64
NTN018	28	Caucasian	Negative	Negative	2	No	3	Yes	No	29
NTN020	74	Caucasian	Unknown	Negative	3	Yes	3	No	No	61
NTN021	56	African American	Unknown	Negative	2	No	3	No	Yes	48
NTN022	66	Caucasian	Unknown	Negative	3	Yes	3	No	No	39
NTN023	31	Caucasian	Negative	Negative	3	Yes	3	Yes	No	63
NTN024	74	Caucasian	Unknown	Negative	2	No	2	Yes	No	49
NTN025	53	African American	Negative	Negative	2	No	3	Yes	No	62
NTN028	40	Caucasian	Negative	Negative	3	Yes	3	No	Yes	39
NTN029	50	African American	Negative	Negative	2	Yes	3	No	No	61
NTN031	46	Caucasian	Negative	Negative	3	Yes	3	Yes	No	56
NTN033	31	Caucasian	Negative	Negative	2	Yes	3	No	Yes	24
NTN034	27	African American	BRCA1	BRCA1	2	No	3	Yes	No	29
NTN037	58	African American	Unknown	Negative	2	No	3	Yes	No	44
NTN038	42	Caucasian	Unknown	Negative	2	Yes	3	No	No	53
NTN039	51	African American	Negative	Negative	2	No	3	Yes	No	28
NTN040	51	Caucasian	Unknown	Negative	2	Yes	2	No	No	54
NTN042	65	African American	Negative	Negative	2	No	2	No	No	53
NTN044	64	Caucasian	Unknown	Negative	2	No	3	Yes	No	54
NTN046	49	Caucasian	Unknown	Negative	2	Yes	3	Yes	No	42
NTN049	46	Caucasian	Negative	Negative	2	Yes	2	No	No	50
NTN050	59	Caucasian	Negative	Negative	2	No	3	Yes	No	41
NTN052	63	Caucasian	Unknown	Negative	2	No	3	No	No	43
NTN056	54	Caucasian	Unknown	Negative	2	Yes	2	No	No	41
NTN057	53	Caucasian	Negative	Negative	2	No	3	No	No	47
NTN058	40	Caucasian	Negative	Negative	2	No	3	Yes	Yes	19
NTN060	25	Caucasian	Unknown	Negative	2	No	3	Yes	No	20
NTN062	64	African American	Unknown	Negative	2	Yes	3	No	No	28
NTN063	59	Caucasian	Negative	Negative	2	No	3	No	No	47
NTN069	55	Caucasian	Unknown	Negative	2	No	3	No	No	45
NTN074	70	Caucasian	Unknown	Negative	2	No	3	No	No	43
NTN076	28	African American	BRCA1	BRCA1	2	Yes	3	No	Yes	41
NTN080	34	Other	Negative	Negative	3	Yes	3	No	No	27
NTN081	56	Caucasian	Unknown	Negative	3	Yes	3	No	No	38
NTN083	30	Caucasian	Negative	Negative	2	No	3	Yes	No	37
NTN084	44	Caucasian	Negative	Negative	2	Yes	3	No	No	35
NTN085	48	Caucasian	Negative	Negative	2	No	3	No	Yes	35
NTN087	59	African American	Unknown	Negative	2	No	3	No	No	33
NTN089	69	Caucasian	Unknown	Negative	2	No	3	No	No	35
NTN090	71	African American	Unknown	Negative	2	No	3	No	Yes	30
NTN091	60	African American	Negative	Negative	2	Yes	3	No	No	27
NTN092	67	Caucasian	Unknown	Negative	2	No	3	No	No	19

*Whole exome sequencing (WES) analysis on *BRCA1*/*BRCA2* variant status is provided in Supplementary Table 2.

Dx = diagnosis; pCR = pathological complete response.

### Whole exome sequencing for custom ctDNA micro-panel development

**Figure 3:**
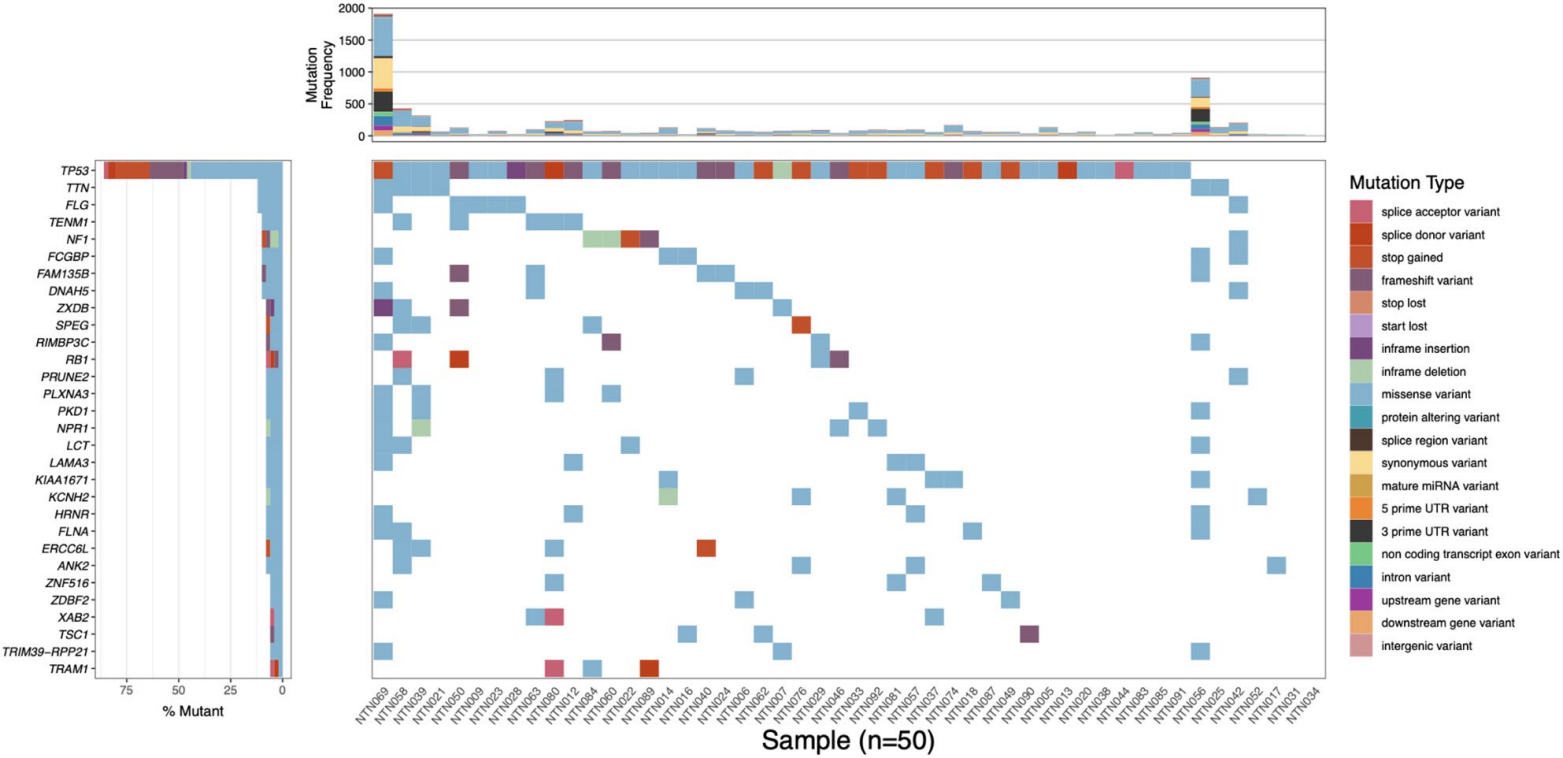
Waterfall plot of Baseline (T0) WES for tumor/matched normal genomic DNA. Each column represents a sample that had whole exome sequencing. Rows represent mutated genes across all samples. Variants predicted to have no impact on protein sequence are only shown in the top mutation frequency panel. The left panel indicates the number of samples containing a mutation in the indicated gene, whereby the top 30 genes are displayed. The top panel indicates the total number of variants observed in each sample, even those that are not shown in the waterfall plot.

Whole exome sequencing was performed on baseline normal and paired tumor samples as well as additional timepoints where available (see Methods). Across all targeted exon positions, the median coverage of normal and tumor samples was 79 bases (range = 44–146 bases) and 73 bases (range = 18–137 bases), respectively. A median of 77.5 somatic variants (range of 1–1970) was identified across all tumors (Fig. 3). In total, 7513 variants were identified. *TP53* mutations were identified in 86% of tumors (n = 43 patients). Two samples (NTN069 and NTN056) had a high mutational burden (> 750 variants); neither patient had an observed BRCA1 / BRCA2 variant (Table 1). WES variants from Baseline (T0) samples (matched tumor/normal tissue biopsies) were used to develop SWIFT custom micro-panels for individual patients. In total, 208 variants were selected across all 50 patients for developing the custom ctDNA micro panels (Supplementary Table 1).

There were 8 samples that had WES of the tumor tissue and custom ctDNA micro-panel sequencing of the peripheral blood at baseline (T0). Across the 33 detectable variants in the 8 samples, 82% (n = 27 variants) were detectable in both the tissue and the peripheral blood, which confirmed accuracy and concordance between tumor sequencing and peripheral blood sequencing. The mean VAF of variants in the baseline tumor tissue was 32.9% whereas the mean VAF of variants in the peripheral blood was 0.33%.

### ctDNA monitoring and clinical outcomes

**Figure 4:**
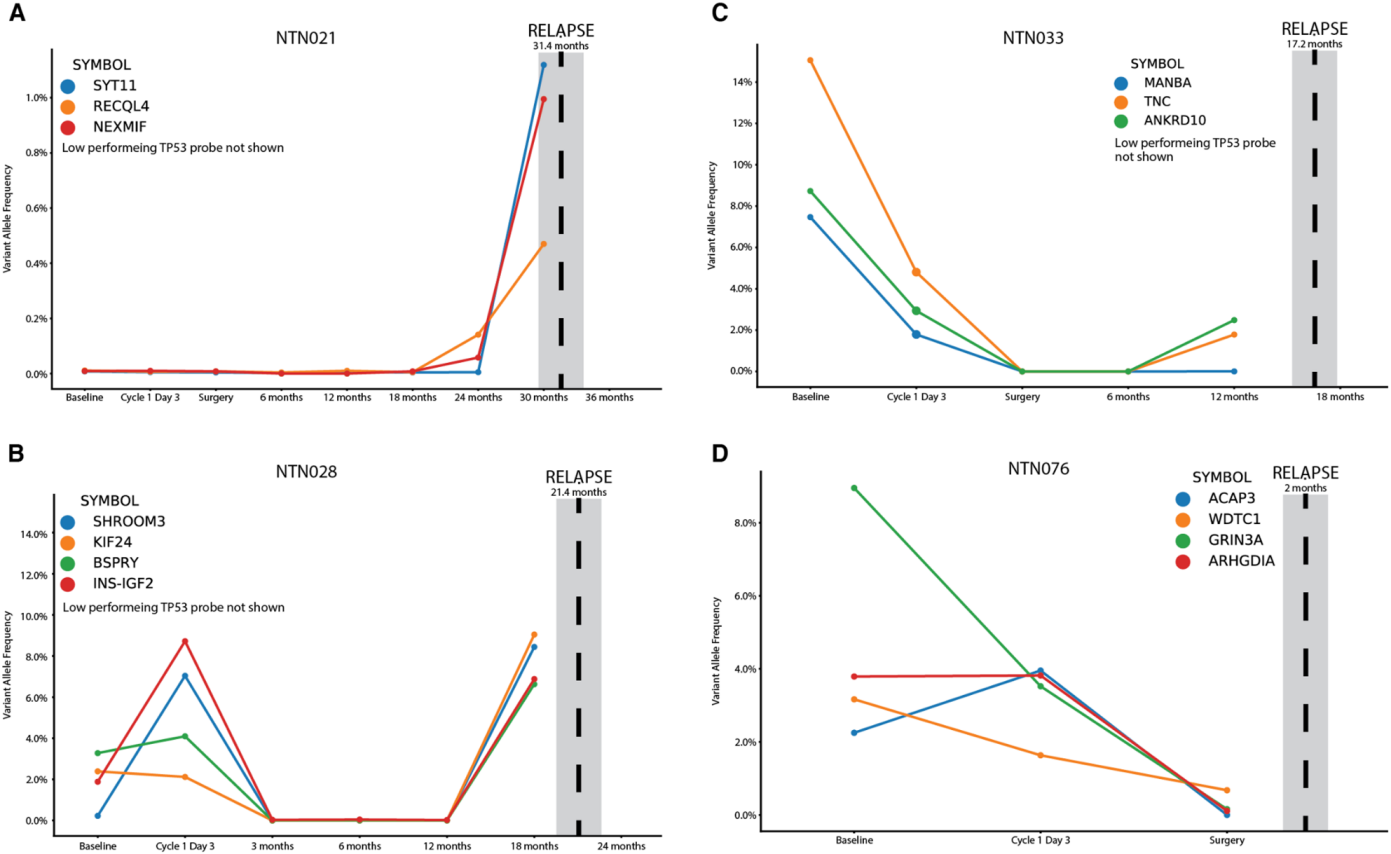
Patients who demonstrated molecular relapse in advance of clinical relapse. Each panel shows VAF for patient-specific tumor markers across various timepoints. Clinical relapse is denoted with a vertical dotted line. Low-performing probes (< 1,000X observed coverage) were not shown. (**A**) NTN021 showed no signal at baseline or at C1D3. A spike in patient-specific VAFs occurred at 24 months, which was 7 months prior to clinical relapse. (**B**) NTN028 showed evidence of patient-specific VAFs at baseline with an increase in VAF at C1D3. The VAF of patient-specific markers were undetectable from 3 months until 12 months with a spike in VAF at 18 months, which was 3 months prior to clinical relapse. (**C**) NTN033 showed patient-specific VAFs at baseline and at C1D3. Patient-specific markers were undetectable at time of surgery and at 6 months. Patient-specific markers spiked at 12 months, which was 5 months prior to clinical relapse. (**D**) NTN076 showed non-zero VAFs of patient-specific markers at the time of surgery with clinical relapse 2 months post-operation.

In total, 208 unique variants were targeted for all 50 unique patient samples (Supplementary Table 1). On average, 4 amplicons were designed for each sample (range = 1–6 amplicons). Of the 50 patients analyzed in this study, 7 patients had a clinical relapse reported during the disease monitoring period. Of these, 4 had ctDNA sequencing performed within the 6 months of reported relapse (Fig. 4). Three of the four patients showed molecular recurrence of the patient-specific amplicon signature prior to reported clinical recurrence (Fig. 4A–C) and an additional patient showed persistent/residual disease at the time of surgery prior to relapse at 2 months post-surgery (Fig. 4D). There were 3 additional patients with clinical relapse, however their most recent ctDNA timepoint was greater than 6 months prior to relapse, precluding the ability of the ctDNA micro-panel in predicting clinical relapse (Supplementary Fig. 1). These data indicate that molecular relapse using the ctDNA micro-panel detected 4 of 4 patients in advance of clinical relapse if the ctDNA micro-panel was performed within 6 months of clinical relapse.

**Figure 5:**
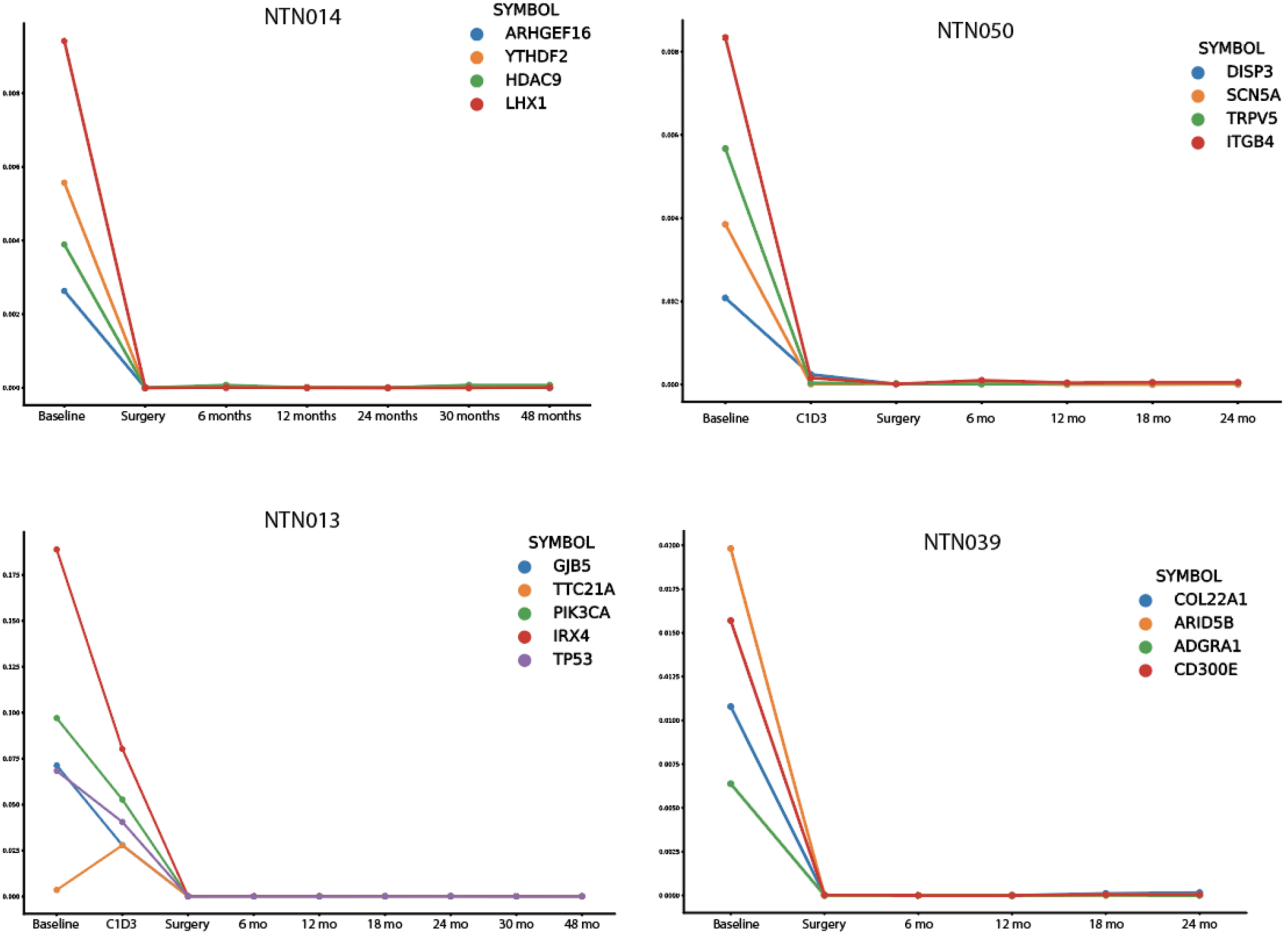
Monitoring clinical remission using a ctDNA molecular signature. Each panel shows patient-specific VAFs across various timepoints. In total there were 17 patients that had both molecular and clinical remission. A representative 4 patients are shown below. The remaining 13 patients are shown in Supplementary Fig. 2.

Twenty-one patients did not develop clinical relapse during monitoring. Of these, 17 showed molecular remission using the custom ctDNA micro-panel (Fig. 5, Supplementary Fig. 2). There were 4 patients who demonstrated potential molecular relapse with no clinical relapse (Supplementary Fig. 3). One patient (NTN0022) was lost to follow up within the conceivable timeframe of clinical relapse. The other 3 patients were followed for at least 22 months post potential molecular relapse. Given that only one of the 3–4 variants for each sample was detected as positive at the molecular level, it is possible that these samples were potentially false positives. These patients are still being followed on the parent trial.

Of the 50 patients analyzed in this study, 22 were not discussed above: 16 patients had either no baseline data, no data post-surgery, or insufficient biomarkers for assessment (Supplementary Fig. 4); 4 patients had a ctDNA VAF of baseline variants at < 0.05% (Supplementary Fig. 5A), and 2 patients had variants that appeared to be germline variants (e.g., detected at 100% VAF in peripheral blood) (Supplementary Fig. 5B).

### Association between ctDNA positivity and pCR

**Figure 6:**
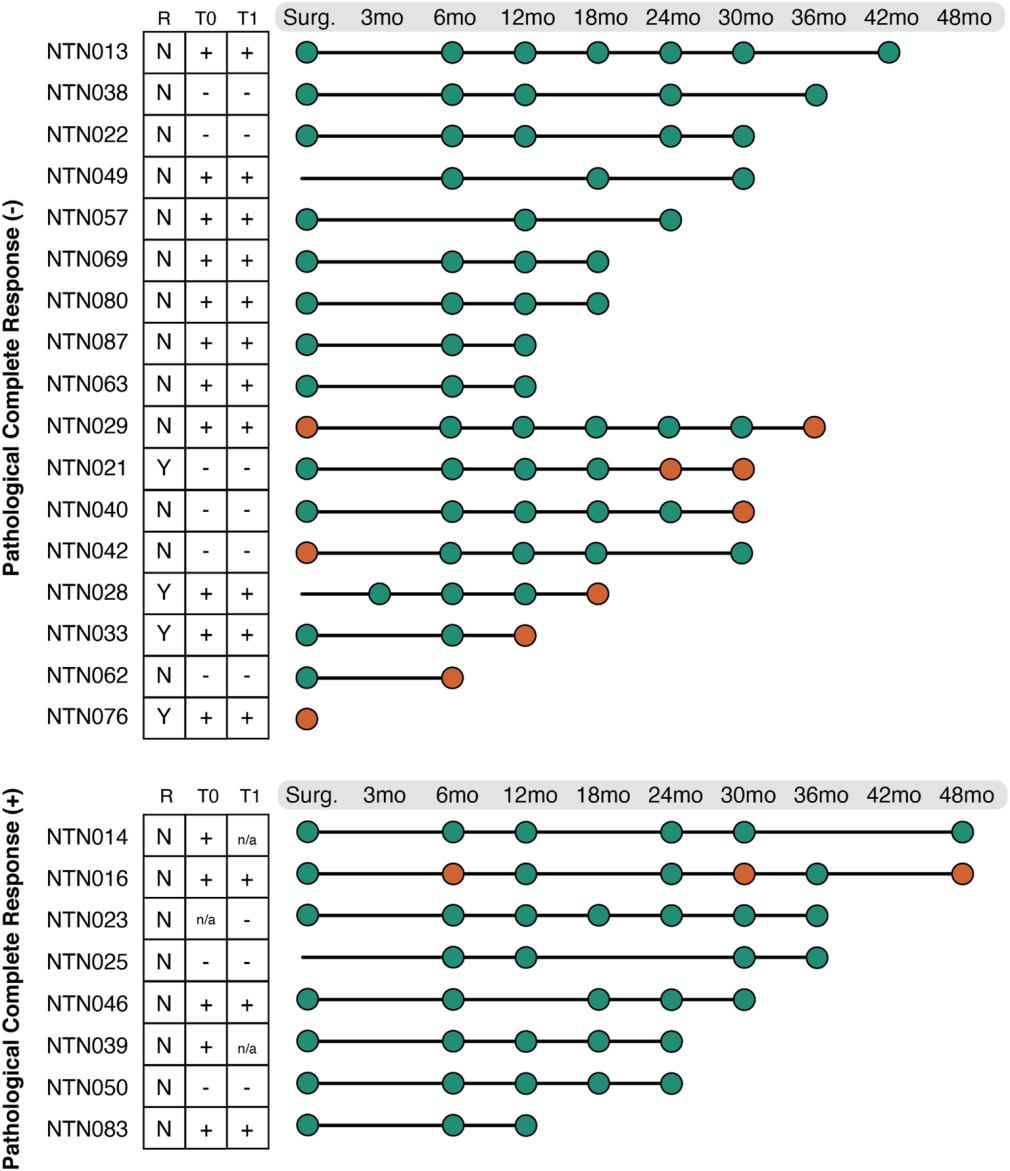
Association between molecular relapse (tumor-specific VAF detection) and pathological Complete Response (pCR). An algorithm was created to assess association between pCR and observed molecular relapse using ctDNA VAF detection. The algorithm was considered positive if any of the variants in the custom micro-panel had a VAF > 0.005%. Each patient was assessed at each timepoint to determine positivity. If the ctDNA demonstrated molecular relapse, the timepoint was labeled in orange; ctDNA timepoints with no molecular relapse were labeled in green. Abbreviations: R = Clinical Remission; T0 = Baseline; T1 = Cycle 1 Day 3 (C1D3).

Of the 50 patients analyzed in this study, 25 were eligible for evaluating ctDNA as a biomarker of recurrence (Figs. 4, 5, Supplementary Fig. 2–3). A sample was determined to have a positive ctDNA result if any variant in the custom ctDNA micro-panel had a VAF > 0.005%. Each patient was assessed at each timepoint to determine positivity. Of the 8 individuals with pCR, only 1 patient (12.5%) showed a positive ctDNA result at any timepoint during remission. Conversely, of the 17 individuals without pCR, 8 patients (47%) showed a positive ctDNA result for at least one timepoint during remission. The difference in the positivity rate between patients with pCR and patients without pCR showed non-significant but trending correlation (*p*-value = 0.10; Chi-Square test) (Fig. 6).

## Discussion

This observational study demonstrated that a custom micro-panel of 4–6 patient-specific variants applied to ctDNA could be used to predict and monitor disease recurrence for patients with early-stage TNBC. Each variant in this custom panel was unique to the individual, allowing for a precision approach for identifying molecular relapse in advance of clinical relapse. Detection of patient-specific variants in ctDNA also showed trending correlation with failure to achieve pCR, suggesting potential prognostic utility in patients with TNBC.

The use of a custom ctDNA micro-panel provided possible improvements relative to traditional universal panels that have a set number of genes/loci for all tumors being evaluated. Specifically across the 50-patient cohort, we identified over 200 variants derived from 177 unique genes, and 204 unique genomic loci. Use of a universal panel might not cover useful variants that provide adequate sensitivity for detection of molecular relapse during surveillance, which were potentially informative of clinical correlates such as molecular relapse and association with pCR.

We demonstrated that assessment for residual disease by ctDNA is possible at approximately 6 month intervals. While large comprehensive panels could be prohibitively expensive for assessing residual disease at high-frequency intervals, smaller ctDNA micro-panels might be more feasible for confirming molecular remission. Additionally, since the custom micro-panels are directly associated with the patient’s tumor, using these patient-specific variants might improve sensitivity of molecular recurrence in advance of clinical relapse.

Custom variants identified for each individual’s tumor were not required to be known oncogenes or tumor suppressors, however, many of the variants identified via WES (Fig. 3) and ctDNA (Fig. 4) had clinical significance. *TP53* contained the highest number of variants across all tumors, which has implications related to prognosis in TNBC^43^. Additionally, putative driver variants in *NF1*^44,45^ and *RB1*^46^, have demonstrated clinical relevance in previously reported breast cancer clinical trials. Interestingly, genes that are rarely mutated across all TNBCs, including *RECQL4*^47^ and *TNC*^48^, were observed in some patients in this study and have potential clinical significance related to prognosis and therapeutic response. Use of a custom panel to assess TNBC during disease monitoring allows providers to assess and potentially select for clinically-relevant variants that are unique to an individual’s tumors further providing advantages against a universal approach.

WES at baseline permitted confirmation of *BRCA1* / *BRCA2* variants observed in patients with TNBC. Three patients had confirmed germline predisposition status and 22 patients with unknown status had no observed pathogenic BRCA variants (Supplementary Table 2). The use of baseline WES panels could serve as a replacement for germline predisposition testing while also providing the custom targets for disease monitoring post-resection. This would also mitigate the need for multiple small panels for germline predisposition testing.

There were some limitations to this study. First, there were several patients who were lost to follow-up (see Table 1). Additionally, half of the patients (*n* = 25) had no peripheral blood for ctDNA isolation collected at timepoints that were imperative for analysis, preventing them from being part of the primary analysis. For example, 3 of the patients who developed clinical recurrence had a blood collection at more than 7 months prior to recurrence, potentially preventing the ability to detect recurrence at the molecular level. Lack of study compliance with required protocols greatly impacted the ability to detect significance for findings. Additionally, peripheral blood collected post-relapse would have been important to demonstrate the VAF threshold for clinical remission. Finally, it was observed that some probes did not generate sufficient coverage in peripheral blood indicating the need for more than 1–2 variants in the micro-panel and rationale for why some other studies with smaller panels demonstrated limited clinical utility^49^. We also recognize that post-surgery treatments were at treating physician's discretion and some of these treatments may have impacted detection of ctDNA.

Future studies are necessary to further demonstrate the use of personalized tumor micro-panels to predict and monitor TNBC remission. Additional timepoints with more frequent intervals for ctDNA testing could be utilized to further refine the sensitivity of the approach to detect molecular recurrence. Developing methods of accurately identifying patients with TNBC who are at a high risk of relapse is an unmet medical need. If clinically accurate biomarkers are developed, interventions that may change the natural history of relapsed TNBC could also be developed.

## Supplementary Information

Supplementary Information 1.

Supplementary Information 2.

Supplementary Information 3.

## Data Availability

The datasets generated and/or analyzed during the current study are available in the dbGaP repository (accession: phs002505).
