# Supplementary material for: Personalized ctDNA micro-panels can monitor and predict clinical outcomes for patients with triple-negative breast cancer
Source: Sci Rep. 2022 Oct 22;12:17732. doi: 10.1038/s41598-022-20928-8 (PMC9588015; doi:10.1038/s41598-022-20928-8)

Supplementary Figure 1. Patients whereby clinical relapse was not detected due to lack of sequencing data at least 6 months prior to relapse time point. Each panel shows VAF for patient-specific tumor markers across various time points. Clinical relapse is denoted with a vertical dotted line. Hash marks indicate that the most recent ctDNA time point was greater than 6 months from clinical relapse.

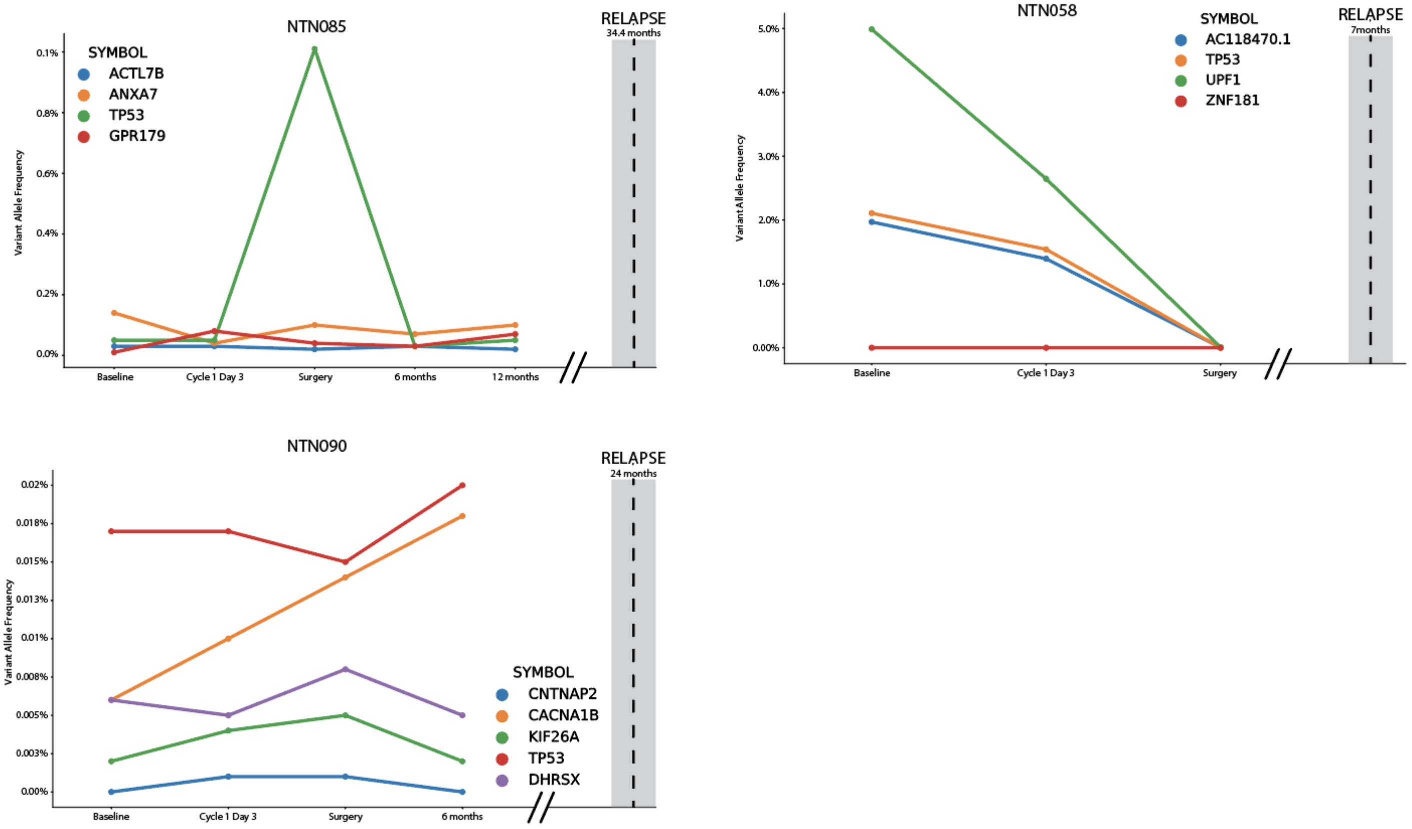

Supplementary Figure 2. Monitoring remission using molecular signature. Each panel shows VAF for patient-specific markers across various time points. All 13 patients shown below were in both molecular and clinical remission at the time of study conclusion. Representative patients are shown in **Figure 5**.

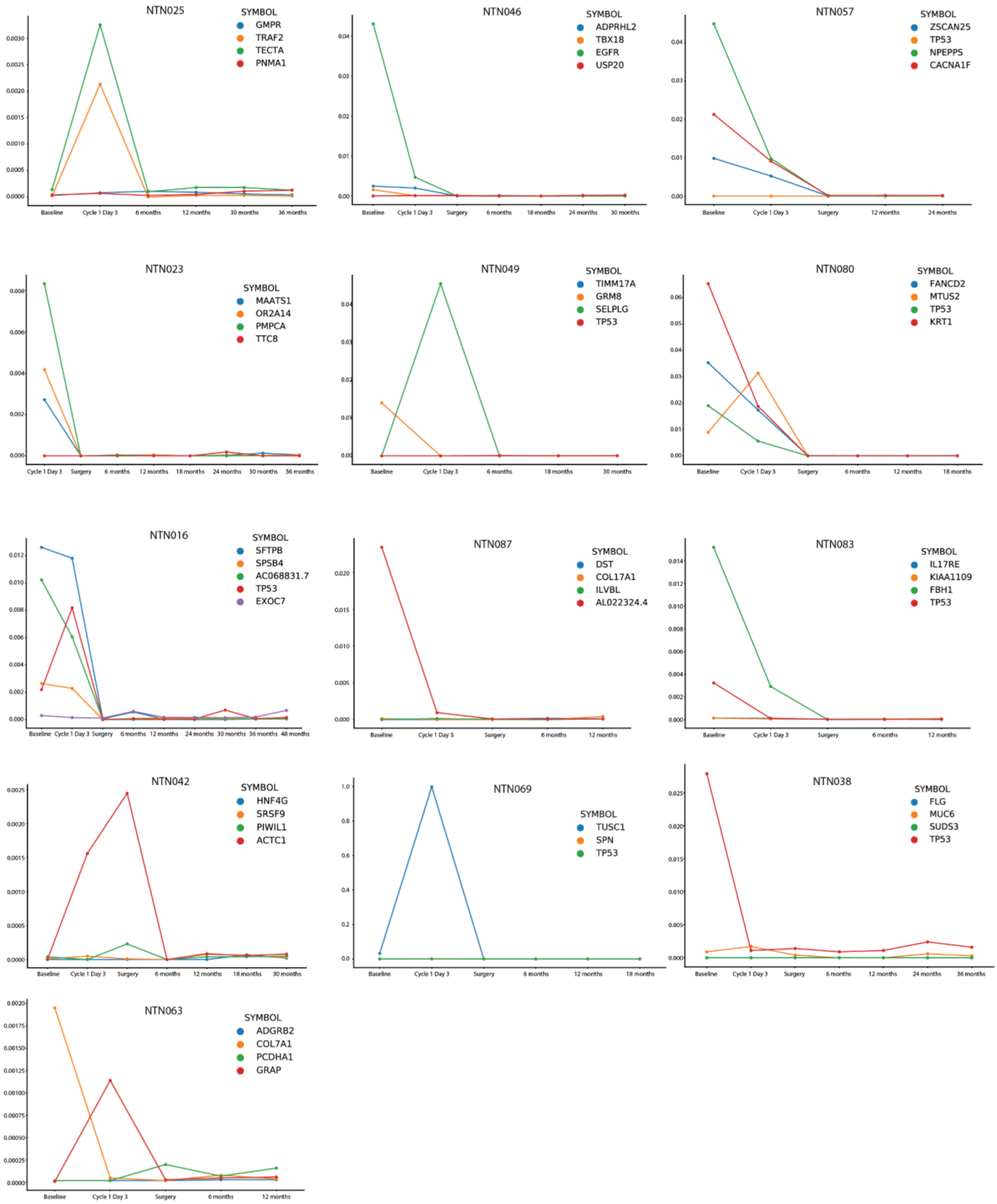

Supplementary Figure 3. Patients with potential molecular relapse with no clinical relapse. Each panel shows VAF for patient-specific tumor markers across various time points. For each of the four cases, one or more variant(s) associated with the ctDNA micro-panel was present relative to the most previous time point. Most recent follow up time was indicated.

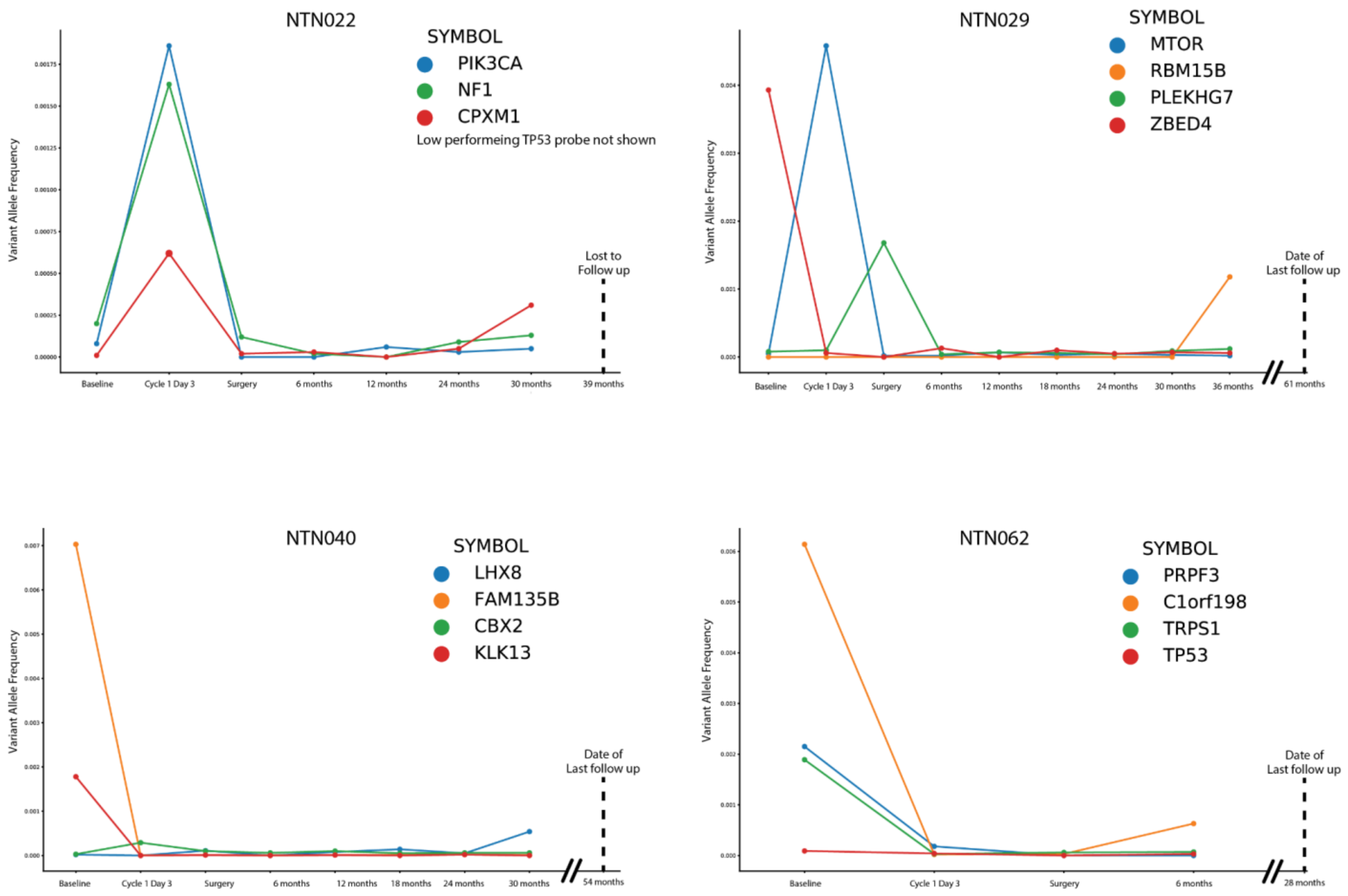

Supplementary Figure 4. Patients with insufficient sequencing data to evaluate the clinical utility of custom micro-panel. Four samples had only a single time point. 9 samples had no data post-surgery. Two samples had no baseline data. One sample had insufficient biomarkers for assessment.

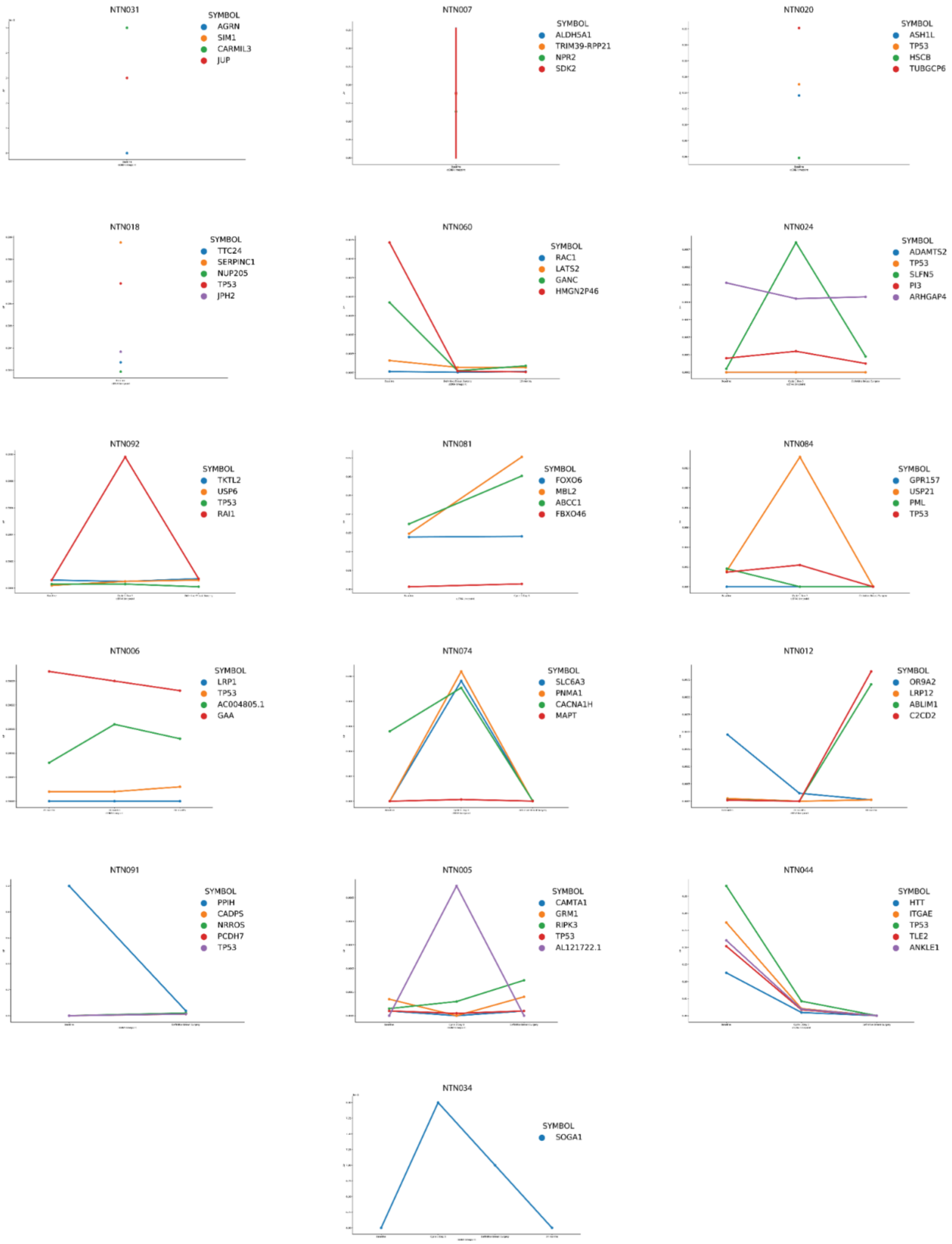

Supplementary Figure 5. There were six patients that could not be analyzed due to either peripheral detection below a minimal level of detection (VAF<0.05%) or due to micro-panels not being associated with the patient's tumor. A). Four patients had a peripheral VAF for all variants at <0.05% **B)** Two patients had variants that were not specific to the tumor. The first patient showed alterations in VAF across all timepoints and the second patient showed that variants were potential germline variants with ~100% VAF at multiple time points.

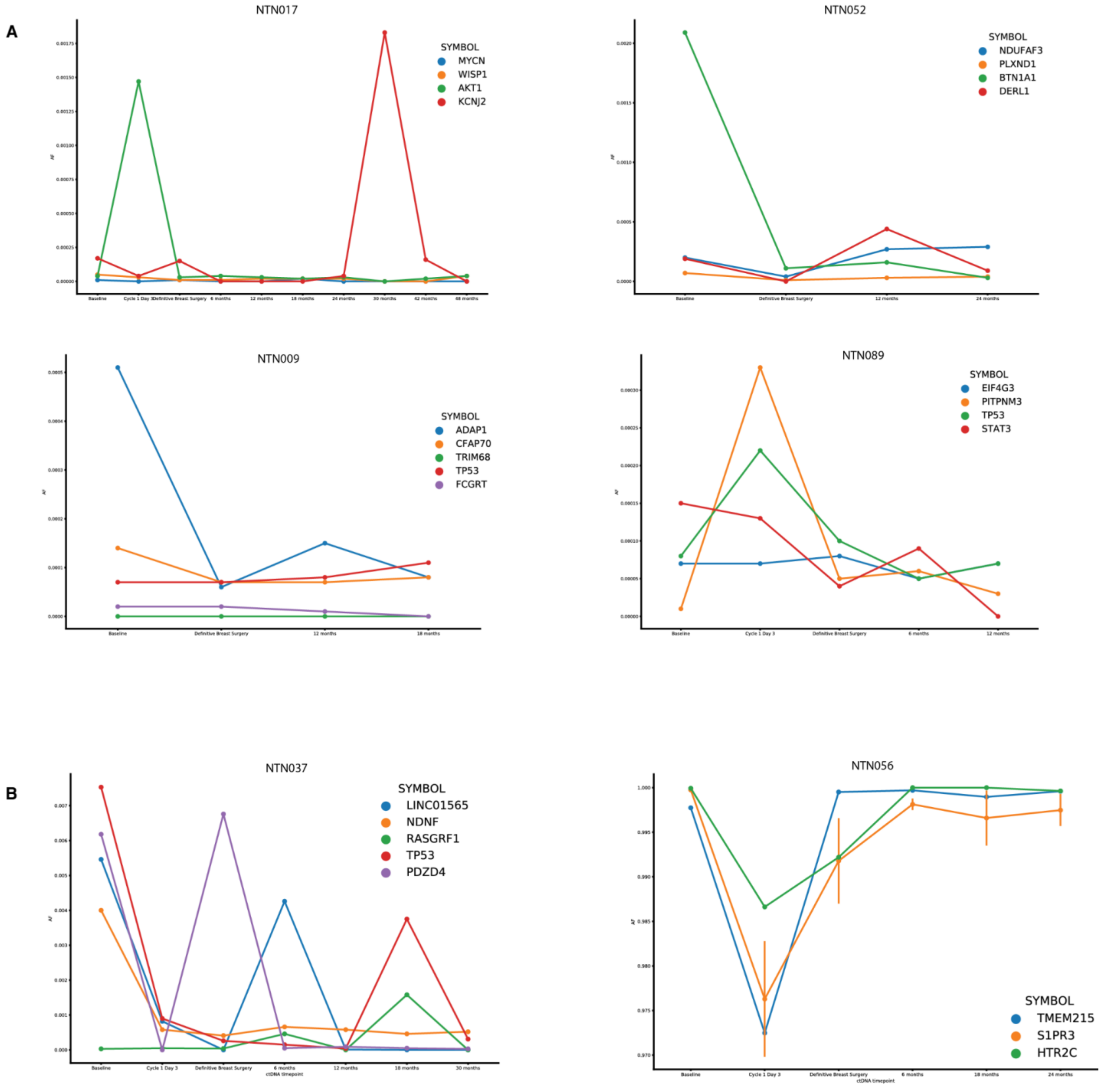

Supplement: Supplementary file 1 — Supplementary Information 1. [file 41598_2022_20928_MOESM1_ESM.pdf]
